# A note on retrograde gene transfer efficiency and inflammatory response of lentiviral vectors pseudotyped with FuG-E vs. FuG-B2 glycoproteins

**DOI:** 10.1038/s41598-019-39535-1

**Published:** 2019-03-05

**Authors:** Soshi Tanabe, Shiori Uezono, Hitomi Tsuge, Maki Fujiwara, Miki Miwa, Shigeki Kato, Katsuki Nakamura, Kazuto Kobayashi, Ken-ichi Inoue, Masahiko Takada

**Affiliations:** 1 Systems Neuroscience Section, Department of Neuroscience, Primate Research Institute, Kyoto University, Inuyama, Aichi 484-8506 Japan; 2 Cognitive Neuroscience Section, Department of Neuroscience, Primate Research Institute, Kyoto University, Inuyama, Aichi 484-8506 Japan; 3 Department of Molecular Genetics, Institute of Biomedical Sciences, Fukushima Medical University School of Medicine, Fukushima, Fukushima 960-1295 Japan; 4 PRESTO, Japan Science and Technology Agency, Kawaguchi, Saitama 332-0012 Japan

## Abstract

Pseudotyped lentiviral vectors give access to pathway-selective gene manipulation via retrograde transfer. Two types of such lentiviral vectors have been developed. One is the so-called NeuRet vector pseudotyped with fusion glycoprotein type E, which preferentially transduces neurons. The other is the so-called HiRet vector pseudotyped with fusion glycoprotein type B2, which permits gene transfer into both neurons and glial cells at the injection site. Although these vectors have been applied in many studies investigating neural network functions, it remains unclear which vector is more appropriate for retrograde gene delivery in the brain. To compare the gene transfer efficiency and inflammatory response of the NeuRet vs. HiRet vectors, each vector was injected into the striatum in macaque monkeys, common marmosets, and rats. It was revealed that retrograde gene delivery of the NeuRet vector was equal to or greater than that of the HiRet vector. Furthermore, inflammation characterized by microglial and lymphocytic infiltration occurred when the HiRet vector, but not the NeuRet vector, was injected into the primate brain. The present results indicate that the NeuRet vector is more suitable than the HiRet vector for retrograde gene transfer in the primate and rodent brains.

## Introduction

Gene transfer of functional molecules into neurons that give rise to a particular pathway is crucial for monitoring/manipulating neuronal activity within that pathway. Emphasis was recently placed on the development of viral vectors that allow us to introduce foreign genes retrogradely into a given pathway and dissect it from others. For example, recombinant glycoprotein-deleted rabies virus (RV) and canine adenovirus-2 (CAV-2) vectors have been used not only for network-tracing studies^1–3^ but also for network-manipulation^4–6^ and bioimaging studies^7–9^. However, these vectors are not suitable for chronic electrophysiological studies or gene therapeutic trials because of the cytotoxicity of their viral proteins. To overcome this, it is necessary to create a novel non-cytotoxic vector that accommodates efficient retrograde gene transfer.

Lentiviral vectors derived from human immunodeficiency virus type 1 (HIV-1) are a strong candidate since they exhibit low cytotoxicity because of their replication-defective form^10,11^. Given that these vectors are typically pseudotyped with vesicular stomatitis virus glycoprotein (VSV-G), they are taken up preferentially from somas/dendrites of neurons. To enable retrograde gene transfer, pseudotyping of lentiviral vectors with rabies virus glycoprotein (RV-G) was attempted^12,13^. Later, fusion envelope glycoproteins (FuG) consisting of distinct combinations of RV-G and VSV-G were developed to enhance the efficiency of retrograde transgene delivery^14–19^. In particular, our research group previously reported the development of two types of lentiviral vectors with highly-efficient retrograde gene transfer. One is known as HiRet vector, which is pseudotyped with the FuG-B2 type of fusion glycoprotein formed by the extracellular and transmembrane domains of RV-G and the membrane proximal regions of VSV-G^15,17^. The other is known as NeuRet vector, which is pseudotyped with the FuG-E type of fusion glycoprotein consisting of the N-terminal segment of RV-G and the membrane proximal region and the transmembrane/cytoplasmic domains of VSV-G^19^. Although these lentiviral vectors permit potent tools for pathway-selective gene manipulation in the central nervous systems of rodents^17,20–23^ and primates^24–26^, it is still unclear which vector possesses a higher efficiency of retrograde transgene delivery.

There has been considerable interest in neurotoxic immune responses triggered by viral vectors transducing foreign proteins into antigen-presenting cells like astroglia. In fact, it has been demonstrated that transduction of a foreign protein (i.e., green fluorescent protein) by adeno-associated virus serotype-9 (AAV9) causes inflammation associated with microglial and lymphocytic infiltration^27,28^. Similar to AAV9, the HiRet vector displays gene transfer into both neurons and glial cells at the injection site^15,17^. Transgene delivery into glial cells by the HiRet vector around the injection site might induce an adaptive immune response leading to prominent damage of transduced tissue. On the other hand, the NeuRet vector preferentially transduces neuronal cells and rarely transduces dividing glial and neural stem/progenitor cells^19,29^. This neuronal specificity of the NeuRet vector reduces the risk of tumorigenesis due to prevention of transgene insertion into the genome of dividing cells in the brain. However, the issue of how much of an immune response and/or neuroinflammation the HiRet and NeuRet vectors cause has not as yet been addressed.

The present study examined the suitability of the HiRet vs. NeuRet vectors for retrograde gene transfer in the brain by comparing them in terms of vector production, transgene expression, and inflammatory response. The striatal input system was employed as a test system, and for comparison among different species, one rodent (rats) and two nonhuman primate (macaques and marmosets) species were used. We have found that the NeuRet vector exhibits a higher retrograde gene transfer efficiency and a much weaker inflammatory response than the HiRet vector. This combination of properties makes the NeuRet vector more viable approach to retrograde transgene delivery in the brains of primates and rodents.

## Results

### Production efficiency of the FuG-B2 and FuG-E pseudotyped lentiviral vectors

**Figure 1:**
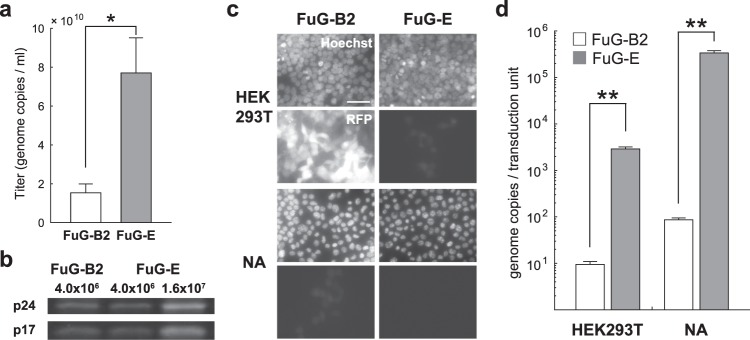
Production efficiency of pseudotyped lentiviral vectors. HEK293T cells in 72 10-cm tissue culture dishes were transfected with the envelope plasmid encoding FuG-E or FuG-B2, together with the transfer plasmid encoding RFP and packaging plasmids. Vector particles were concentrated by centrifugation, purified with ion-exchange chromatography and finally prepared in 110 μl of PBS. (**a**) The yield in the vector stock solutions measured by quantitative reverse transcription-PCR analysis (open bar for FuG-B2, gray bar for FuG-E). Each value was obtained from five individual experiments and expressed as the mean ± SEM. *P < 0.05, significant difference from the FuG-B2 pseudotype (Student’s *t* test). (**b**) Fluorescent images of SDS-PAGE of the viral protein (p24 and p17) from vectors pseudotyped with FuG-B2 (4.0 × 10^6^ genome copies) and FuG-E (4.0 × 10^6^ and 1.6 × 10^7^ genome copies). A full-length gel image is presented in Supplementary Fig. S3. (**c**) Fluorescent images of Hoechst-stained HEK293T and Neuro2A (NA) cells after transduction of the vectors pseudotyped with FuG-B2 and FuG-E (100 genome copies/cell). Scale bar, 50 μm. (**d**) Ratio of the RNA genome copies to the transduction units (open bars for FuG-B2, gray bars for FuG-E). Each value was obtained from five individual experiments and expressed as the mean ± SEM. **P < 0.01, significant difference from the FuG-B2 vector (Student’s *t* test).

We produced HIV-1-based lentiviral vectors carrying the red fluorescent protein (RFP) transgene by pseudotyping with FuG-B2 or FuG-E glycoprotein as reported in previous studies^17,19^. To compare the recovery efficiency of the FuG-E and FuG-B2 pseudotype, the copy number of viral RNA in the vector stock solutions with the same concentration volume was determined by quantitative reverse transcription-PCR (Fig. 1a). The RNA copy number in the vector stock solution of the FuG-E pseudotype was significantly greater than that of the FuG-B2 pseudotype (Student’s *t* test, *P* < 0.05), and this RNA copy number of the FuG-E pseudotype was equivalent to that of the VSV-G pseudotype (Supplementary Fig. S1). SDS-PAGE analysis revealed that the amount of viral protein (p24 and p17) content in the vector solutions adjusted to the same RNA titer (4.0 × 10^6^ genome copies) did not differ between the FuG-E and FuG-B2 pseudotypes (Fig. 1b). This indicates that the vector stock solutions with identical RNA titer contained an equivalent number of viral vector particles. We also found that the higher-titer vector solution (1.6 × 10^7^ genome copies) of the FuG-E pseudotype contained more viral protein than the lower-titer vector solution (Fig. 1b). These results demonstrate that the FuG-E pseudotype has a higher production efficiency than the FuG-B2 pseudotype. As reported in a previous study^17^, the FuG-B2 pseudotype transduced into HEK293T and Neuro2A cells, whereas the FuG-E pseudotype rarely transduced into these cells (Fig. 1c). The ratio of the RNA copy number to transducing units of the FuG-E pseudotype was significantly higher than that of the FuG-B2 pseudotype in HEK293T and Neuro2A cell lines (Student’s *t* test, *P* < 0.01; Fig. 1d). These results led us to compare the properties of the HiRet (FuG-B2) and NeuRet (FuG-E) vectors with the same genome titer, but not the functional titer.

### Retrograde gene transfer of the HiRet and NeuRet vectors in macaque monkeys

**Figure 2:**
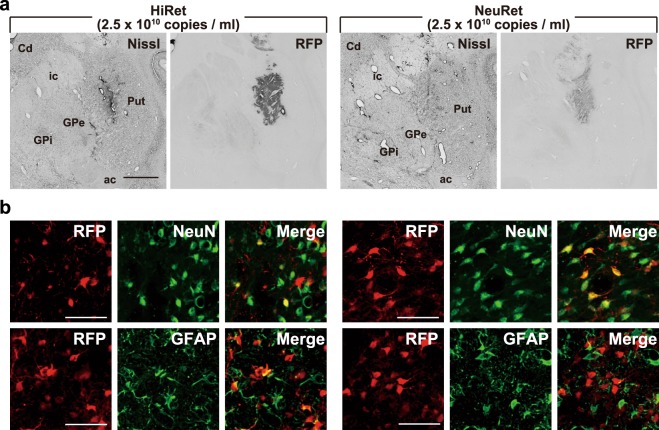
Pattern of gene transfer of the HiRet and NeuRet vectors around injection sites in macaque monkeys. The HiRet and NeuRet lentiviral vectors (each 2.5 × 10^10^ genome copies/ml) were injected into the putamen (Put) of macaque monkeys, and histological analyses were performed in the brains fixed at four weeks postinjection. (**a**) Nissl staining and RFP immunostaining at the injection sites of the HiRet (left) and NeuRet (right) vectors with the same titer. ac, anterior commissure; Cd, caudate nucleus; GPe, external segment of the globus pallidus; GPi, internal segment of the globus pallidus; ic, internal capsule. Scale bar, 2 mm. (**b**) Double immunostaining for RFP (red) and NeuN (upper; green) or GFAP (lower; green) at injection sites of the HiRet (left) and NeuRet (right) vectors. Shown in yellow are double-immunostained cells. Scale bars, 50 μm.

To compare the efficiency of retrograde gene delivery of the HiRet (FuG-B2) and NeuRet (FuG-E) vectors in the brain of macaque monkeys, striatal injections of each vector encoding the RFP gene were made stereotaxically into both the caudate nucleus and the putamen (Fig. 2a). We first injected the HiRet vector (2.5 × 10^10^ genome copies/ml, 60 µl) and the NeuRet vector with an equivalent titer (2.5 × 10^10^ genome copies/ml, 60 µl). Since the production efficacy of the NeuRet vector is higher than that of the HiRet vector, we then injected the NeuRet vector with a higher titer (7.5 × 10^10^ genome copies/ml, 60 µl). To investigate the properties of gene transduction around the injection sites using both vectors with the same titer (2.5 × 10^10^ genome copies/ml), double immunofluorescence histochemistry was performed for RFP and either of the neuronal marker, neuronal nuclei (NeuN), or the astroglial marker, glial fibrillary acidic protein (GFAP) (Fig. 2b). The ratio of RFP + NeuN-positive or RFP + GFAP-positive cells to the total RFP-positive cells was analyzed. In the NeuRet vector injection case, almost all RFP-positive cells co-expressed NeuN (n = 2; 94.1% and 94.3%), while only a few RFP-positive cells co-expressed GFAP (n = 2; 3.2% and 3.5%), as we reported previously^29^. In the HiRet vector injection case, on the other hand, a large number of RFP-positive cells also co-expressed GFAP (n = 2; 32.1% and 28.2%), as well as for NeuN (n = 2; 76.3% and 51.5%). Thus, the HiRet vector exhibited gene transfer into both neurons and glial cells at the injection site, as reported in mice^15,17^, whereas the NeuRet vector showed marked neuronal specificity.

**Figure 3:**
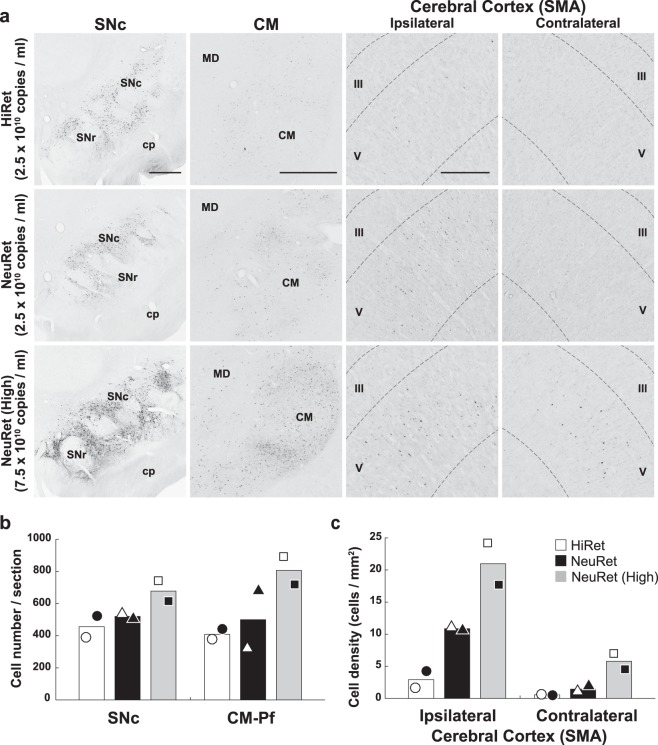
Transgene expression after intrastriatal injections of the HiRet and NeuRet vectors in macaque monkeys. Intrastriatal injections of HiRet (2.5 × 10^10^ genome copies/ml) and NeuRet (2.5 × 10^10^ and 7.5 × 10^10^ genome copies/ml) pseudotyped lentiviral vectors were made in macaque monkeys, and histological analyses were performed in the brains fixed at four weeks postinjection. (**a**) RFP immunostaining in the substantia nigra pars compacta (SNc), centromedian nucleus (CM), and supplementary motor area (SMA) of the ipsilateral and contralateral hemispheres for the HiRet (top), lower-titer NeuRet (middle), and higher-titer NeuRet (bottom) vectors. cp, cerebral peduncle; MD, mediodorsal nucleus; SNr, substantia nigra pars reticulate; III, layer III; V, layer V. Scale bars, 1 mm for SNc and CM, 500 μm for SMA. (**b**) Number of RFP-positive neurons in the SNc and the centromedian-parafascicular complex (CM-Pf). Eleven or seven sections were used for cell counts in the SNc or CM-Pf, respectively. (**c**) Density of RFP-positive neurons in the SMA of the ipsilateral and contralateral hemispheres. Ten sections were analyzed. Each symbol (open or filled circle, triangle, or square) indicates data for each animal, and the same symbol represents data for the same animal. Averaged data (n = 2) are denoted by open (for the HiRet vector), filled (for the NeuRet vector with lower titer), and gray (for the NeuRet vector with higher titer) bars.

To compare the efficacy of retrograde gene delivery of the HiRet and NeuRet vectors with the same titer (2.5 × 10^10^ genome copies/ml), we analyzed the density of neuronal labeling in the substantia nigra pars compacta (SNc), the centromedian-parafascicular thalamic complex (CM-Pf), and the supplementary motor area (SMA). In the SNc and CM-Pf, somewhat larger numbers of RFP-positive neurons were found after the injection of the NeuRet than the HiRet vector (Fig. 3a). The average number of RFP-positive neurons transduced with the HiRet or NeuRet vector in the SNc was 456.2 or 520.6 cells per section (n = 2), respectively (Fig. 3b; NeuRet/HiRet ratio = 1.1), while that of RFP-positive CM-Pf neurons in the HiRet or NeuRet vector injection case was 408.7 or 500.0, respectively (Fig. 3c; NeuRet/HiRet ratio = 1.2). Double immunofluorescence histochemistry for RFP and the dopaminergic neuron marker, tyrosine hydroxylase (TH), confirmed that substantially all RFP-positive neurons in the SNc were also immunostained for TH, and nearly half of the total TH-immunostained neurons expressed the RFP transgene in the NeuRet (46.3%; n = 2) and HiRet (43.5%; n = 2) vector injection cases. On the other hand, the HiRet and NeuRet vectors with the same titer (2.5 × 10^10^ genome copies/ml) transduced only a small number of neurons in the SMA, though neuronal labeling with the NeuRet vector was still noticeable in the ipsilateral hemisphere (Fig. 3a). However, a large number of labeled neurons were observed not only in the ipsilateral but also in the contralateral SMA after the injection of the NeuRet vector with a higher titer (7.5 × 10^10^ genome copies/ml). The density of RFP-positive neurons in the case of injecting the higher-titer NeuRet vector was greater than that in the case of the lower-titer NeuRet vector by approximately 1.9 or 4.0 times in the ipsilateral or contralateral SMA, respectively (Fig. 3c).

### Retrograde gene transfer of the HiRet and NeuRet vectors in marmosets

**Figure 4:**
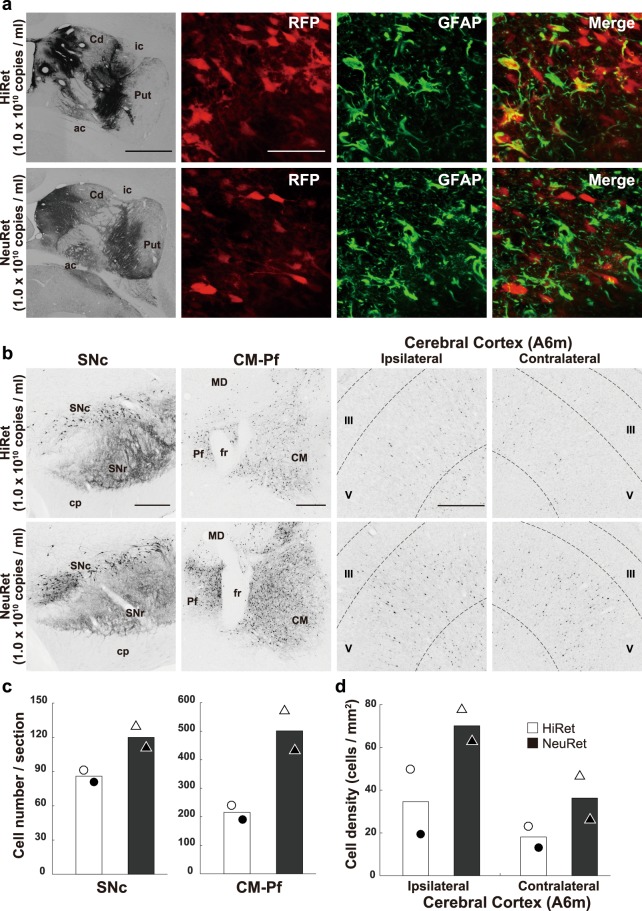
Transgene expression after intrastriatal injections of the HiRet and NeuRet vectors in marmosets. The HiRet and NeuRet vectors (each 1.0 × 10^10^ genome copies/ml) were injected into the Cd and Put of marmosets, and histological analyses were performed in the brains fixed at four weeks postinjection. (**a**) Injection sites of the HiRet (upper; gray) and NeuRet (lower; gray) vectors and cells double-immunostained for RFP (red) and GFAP (green) at the injection sites. Shown in yellow are double-immunostained cells. Scale bars, 1 mm for injection sites, 50 μm for double-immunostained cells. (**b**) RFP immunostaining in the SNc, CM-Pf, and Brodmann’s area 6 m (A6m) of the ipsilateral and contralateral hemispheres for the HiRet (upper) and NeuRet (lower) vectors. The roman numerals in each A6m panel indicate the cortical layers. fr, fasciculus retroflexus; Pf, parafascicular nucleus. Scale bars, 500 μm. (**c**) Number of RFP-positive neurons in the SNc and CM-Pf. Seven or four sections were used for cell counts in the SNc or CM-Pf, respectively. (**d**) Density of RFP-positive neurons in the A6m of the ipsilateral and contralateral hemispheres. Eight sections were analyzed. Each symbol (open or filled circle or triangle) indicates data for each animal, and the same symbol represents data for the same animal. Averaged data (n = 2) are denoted by open (for the HiRet vector) and filled (for the NeuRet vector) bars. Other conventions are as in Figs 2 and 3.

To compare retrograde gene transfer of the HiRet and NeuRet vectors in the brain of common marmosets, we injected each vector with the same titer (1.0 × 10^10^ genome copies/ml, 10 µl) into the striatum (Fig. 4a). For analyzing the extent of glial transduction at the injection site of each vector, double immunofluorescence histochemistry for RFP and GFAP was performed. Consistent with the results obtained in the macaque monkeys, many RFP-positive cells co-expressed GFAP in the HiRet vector injection case, while few RFP-positive cells co-expressed GFAP in the NeuRet vector injection case. We analyzed the density of labeled neurons in the SNc, CM-Pf, and Brodmann’s area 6 m (A6m). In the SNc, many RFP-positive neurons were observed after the NeuRet than the HiRet vector injection (Fig. 4b). The mean number of RFP-positive neurons in the HiRet or NeuRet vector injection case was 86.0 or 120.0 cells per section (n = 2), respectively (Fig. 4c; NeuRet/HiRet ratio = 1.4). Similar, but clearer, data about the superiority of the NeuRet vector were obtained for neuronal labeling in the CM-Pf (Fig. 4b) and A6m (Fig. 4b). The mean number of RFP-positive CM-Pf neurons in the HiRet or NeuRet vector injection case was 215.5 or 501.1 cells per section (n = 2), respectively (Fig. 4c; NeuRet/HiRet ratio = 2.3). The density of RFP-positive A6m neurons in the HiRet or NeuRet vector injection case was 34.6 or 70.1 for the ipsilateral hemisphere (n = 2), respectively (Fig. 4d; NeuRet/HiRet ratio = 2.0), and it was 18.1 or 36.3 for the contralateral hemisphere (n = 2), respectively (Fig. 4d; NeuRet/HiRet ratio = 2.0).

### Retrograde gene transfer of the HiRet and NeuRet vectors in rats

**Figure 5:**
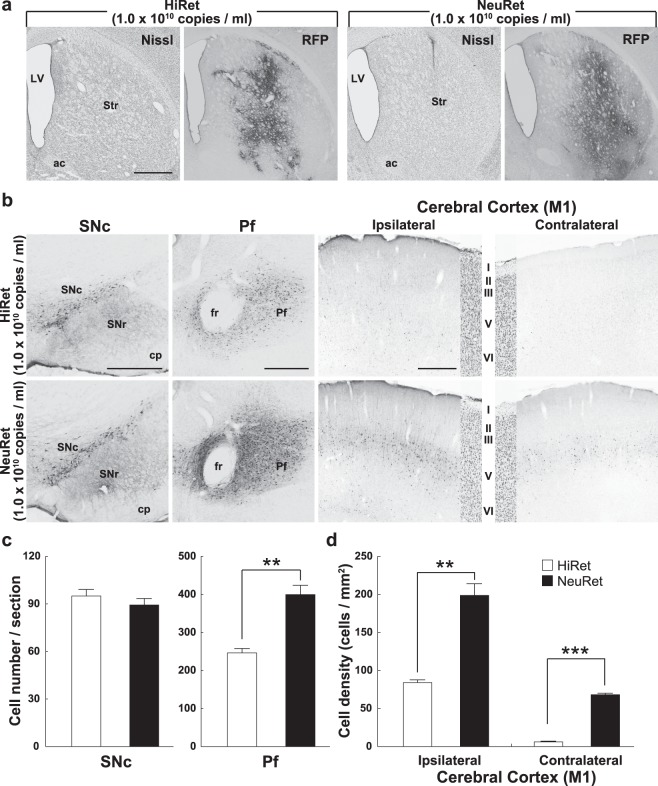
Transgene expression after intrastriatal injections of the HiRet and NeuRet vectors in rats. The HiRet and NeuRet vectors (each 1.0 × 10^10^ genome copies/ml) were injected into the striatum (Str) of rats, and histological analyses were performed in the brains fixed at four weeks postinjection. (**a**) Nissl staining and RFP immunostaining at the injection sites of the HiRet (left) and NeuRet (right) vectors. LV, lateral ventricle. Scale bar, 1 mm. (**b**) RFP immunostaining in the SNc, Pf, and primary motor cortex (M1) of the ipsilateral and contralateral hemispheres for the HiRet (upper) and NeuRet (lower) vectors. In each M1 panel, a Nissl-stained image of the corresponding region is also depicted. Scale bars, 500 μm for SNc, Pf, and M1. (**c**) Number of RFP-positive neurons in the SNc and Pf. Six or four sections were used for cell counts in the SNc or Pf, respectively. (**d**) Density of RFP-positive neurons in the M1 of the ipsilateral and contralateral hemispheres. Four sections were analyzed. Averaged data (n = 4 for each group; error bar, SEM) are denoted by open (for the HiRet vector) and filled (for the NeuRet vector) bars. **P < 0.01, ***P < 0.001, significant difference from the HiRet vector (Student’s *t* test). Other conventions are as in Figs 2–4.

To compare the extent of retrograde gene transfer of the HiRet and NeuRet vectors in the rat brain, we injected each vector with the same titer (1.0 × 10^10^ genome copies/ml, 2 µl) into the striatum (Fig. 5a). We analyzed the number and density of neuronal labeling in the SNc, Pf, and the primary motor cortex (M1). In the SNc, the equivalent number of RFP-positive neurons was observed after the injections of the HiRet and NeuRet vectors (Fig. 5b). The mean number of RFP-positive neurons in the SNc was 95.0 ± 2.7 or 89.3 ± 4.8 cells per section (n = 4) in the HiRet or NeuRet vector injection case (Student’s *t* test, *P* = 0.341; Fig. 5c; NeuRet/HiRet ratio = 0.9). On the other hand, the significant superiority of the NeuRet vector was obtained for neuronal labeling in the Pf and M1 (Fig. 5b). The average numbers of RFP-positive neurons in the Pf were 246.1 ± 11.1 or 399.5 ± 24.3 cells per section (n = 4) in the HiRet or NeuRet vector injection case (Student’s *t* test, *P* < 0.01; Fig. 5c; NeuRet/HiRet ratio = 1.6). The density of RFP-positive M1 neurons in the HiRet or NeuRet vector injection case was 84.0 ± 3.7 or 198.7 ± 16.1 for the ipsilateral hemisphere (n = 4), respectively (Student’s *t* test, *P* < 0.01; Fig. 5d; NeuRet/HiRet ratio = 2.4), and it was 6.1 ± 0.6 or 68.2 ± 1.8 for the contralateral hemisphere (n = 4), respectively (Student’s *t* test, *P* < 0.001; Fig. 5d; NeuRet/HiRet ratio = 11.2).

### Neuroinflammatory responses of the HiRet and NeuRet vectors

**Figure 6:**
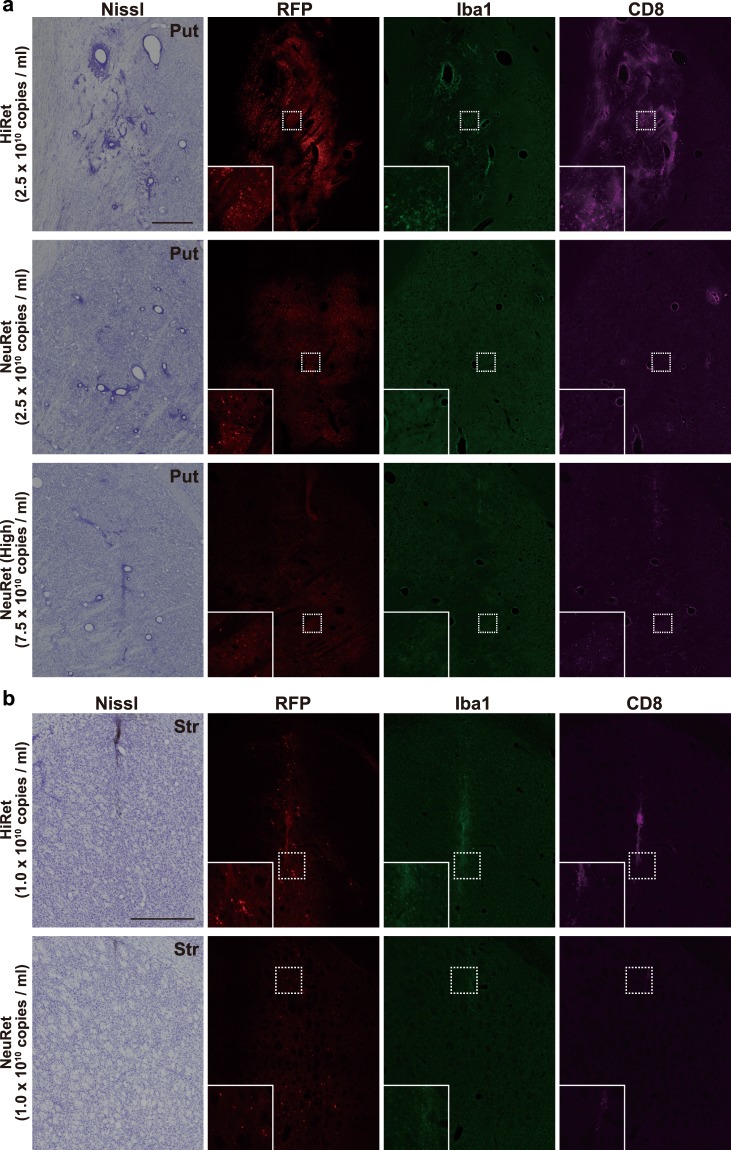
Inflammatory responses after intrastriatal injections of the HiRet and NeuRet vectors in macaque monkeys and rats. (**a**) Nissl staining, RFP-native fluorescence (red), and immunofluorescent staining for Iba1 (green) and CD8 (magenta) at the injection sites of the HiRet (top), lower-titer NeuRet (middle), and higher-titer NeuRet (bottom) vectors in macaque monkeys. Put, putamen, Scale bar, 500 μm. (**b**) Nissl staining, RFP-native fluorescence (red), and immunofluorescent staining for Iba1 (green) and CD8 (magenta) at the injection sites of the HiRet (upper) and NeuRet (lower) vectors in rats. Str, striatum. Scale bar, 500 μm.

As previously reported in mice^17,19^, we have shown that the HiRet vector transduces not only neurons but also glial cells, while the NeuRet vector predominantly transduces neurons in primates. The HiRet vector may thus trigger an immune response due to the introduction of non-self proteins into antigen-presenting cells around the injection site. In Nissl-stained sections from macaque monkeys injected with the HiRet vector, severe necrosis occurred around the injection site (Fig. 6a). To clarify whether this tissue damage related to the immune response, immunofluorescence histochemistry for the microglial marker, ionized calcium-binding adapter molecule 1 (Iba1), and the cytotoxic T cell marker, cluster of differentiation 8 (CD8) was performed. Many Iba1- and CD8-positive cells were observed around the injection site in the HiRet vector injection case, indicating that microglial and lymphocytic infiltration appeared. In contrast to the HiRet vector, no marked necrosis around the injection site was observed after the injections of lower or higher titer of the NeuRet vector. In the marmoset striatum, similar necrotic and microglial/lymphocytic infiltrative events were seen in the HiRet vector, but not the NeuRet vector injection case (Supplementary Fig. S2). Following the intrastriatal injections of either vector, no tissue damage was detected in the rat brain (Fig. 6b).

## Discussion

In previous studies^15–17,19^, it has been reported that pseudotyping a lentiviral vector with modified envelope glycoproteins, formed by RV-G and VSV-G, can improve the efficiency of gene transfer through retrograde transport of the vector. This capability of the pseudotyped lentiviral vectors enables us to express a foreign gene in a particular circuit and remove/manipulate the circuit, not only in rodents^17,20–23^ but also in nonhuman primates^24–26^. In our present work, we investigated the efficacy of transgene expression by the HiRet (FuG-B2) and NeuRet (FuG-E) vectors through the intrastriatal injection of each vector in macaque monkeys, marmosets, and rats. First, we showed that the efficiency of retrograde gene delivery of the NeuRet vector is greater than or equal to that of the HiRet vector, though this depended on the pathway or the species examined. It should be noted, however, that injections of the HiRet vector and the low-titer NeuRet vector into the macaque striatum did not sufficiently label SMA neurons through the corticostriatal pathway. As also reported elsewhere^29^, the NeuRet vector with a higher titer resulted in highly efficient retrograde gene transduction in the corticostriatal pathway, as well as in the nigrostriatal and thalamostriatal pathways. Second, our data also showed that the NeuRet vector exhibits a smaller degree of tissue inflammation than the HiRet vector. Thus, the NeuRet vector could provide a powerful and safe tool to investigate the functional roles of a given pathway in both primates and rodents.

The pathway/species-dependent differences observed in gene transfer efficacy highlights a critical issue in pathway-selective gene manipulation. We have shown that injections of the NeuRet vector achieve efficient retrograde gene transduction into nigrostriatal, thalamostriatal, and corticostriatal neurons not only in macaque monkeys (see also Tanabe *et al*., 2017) but also in marmosets and rats. This indicates that regardless of the striatal input system, the NeuRet vector successfully transduces foreign genes in multiple species. Of particular interest is that the pseudotyped lentiviral vectors only poorly induced retrograde gene transfer into nigrostriatal dopaminergic neurons in mice^15,16^. It is therefore important to clarify the mechanisms underlying the pathway/species-dependence in retrograde gene transduction efficiency.

Concerning tissue inflammation, there was a significant difference between the NeuRet and HiRet vectors. The former preferentially transduces neuronal cells, whereas the latter induces gene delivery into glial cells around the injection site. This is crucial because a neurotoxic immune response may be caused by transducing foreign proteins into antigen-presenting cells. Recently, it has been demonstrated that the capability of AAV9 to transduce antigen-presenting cells in the central nervous systems of rodents and primates provokes the cell-mediated immune response and neuroinflammation^27,28^. In the present study, we observed immune/neuroinflammatory responses around the injection site when the HiRet vector was injected into primate brains. On the other hand, there was no such event in the case of the NeuRet vector injection. It is of interest that in the rat brain, necrosis was not seen around the injection site not only with the NeuRet vector but also with the HiRet vector, as previously reported in mice^17,23^. These results emphasize the safety of the NeuRet vector, and inversely raise an alert over the use of the HiRet vector especially in the primate brain.

In addition to the pseudotyped lentiviral vectors, there are some other viral vectors that permit retrograde gene transfer in the central nervous system. Recombinant glycoprotein-deleted RV and CAV-2 vectors have so far been utilized as tools for manipulating neuronal activity, for example in optogenetics^4,5^. However, the potential cytotoxicity of these vectors may constrain long-term experiments or clinical applications. It is therefore necessary to develop a novel non-cytotoxic vector. In this regard, RV vectors with low cytotoxicity, such as self-inactivated RV vector^30^ and double-deletion-mutant RV vector^31^, have recently been introduced. These RV vectors have been shown to express transgene only transiently and insufficiently, so they rely on Cre- or Flp-mediated recombination and require other helper viral vectors or reporter conditional mouse lines. In addition, a new variant of adeno-associated viral vector termed rAAV2-retro has been developed to induce transgene expression robustly in a retrograde fashion^32^. However, this vector has been reported to have stronger tropisms for particular cell types in mice than the double-deletion-mutant RV vector^31^. Moreover, a major disadvantage of the AAV vector is the limited packaging size of transgene (<5 kb)^33^, compared with the packaging size of lentiviral vectors (<7.5 kb)^34^. To identify the most appropriate vector for retrograde gene transfer, further investigations are needed to compare the NeuRet vector with the above vectors from various perspectives, such as their efficiency, cytotoxicity, and the pathways involved.

The HIV-1-based lentiviral vector has been utilized for pathway-selective gene manipulation^17,20–26^ and gene therapeutic approaches to Parkinson’s disease in rodents and nonhuman primates^35–38^. Our study demonstrates that the NeuRet vector achieves a higher efficiency of gene delivery than the HiRet vector in the primate and rodent brains. Moreover, the NeuRet vector induces immune/inflammatory responses to a lesser extent than does the HiRet vector. This property of the NeuRet vector is ascribable to its high neuronal specificity, which minimizes adverse side-effects such as neuronal loss adjacent to the injection site. In conclusion, the NeuRet vector pseudotyped with FuG-E might gain easy access to successful pathway-specific gene manipulations or effective gene therapeutic trials against neurological disorders such as Parkinson’s disease through improved and safe retrograde delivery.

## Methods

### Animals

Ten adult nonhuman primates of either sex and eight adult male Wistar rats (250–350 g) were used for this study. Of the 10 nonhuman primates, six animals were macaque monkeys (four Japanese monkeys, 5.6–6.2 kg; two rhesus monkeys, 5.6–6.2 kg), and four animals were common marmosets (260–360 g). The experimental procedures were in accordance with protocols approved by the Animal Welfare and Animal Care Committee of the Primate Research Institute, Kyoto University (Permission Number: 2015-033), and were conducted in line with the Guidelines for Care and Use of Nonhuman Primates established by the Primate Research Institute, Kyoto University (2010). Since the Guidelines require us to consider 3R-type recommendations in designing monkey experiments, we decided to use two animals for each purpose. All experiments were performed in a special laboratory (biosafety level 2) designated for *in vivo* animal infectious experiments that had been placed at the Primate Research Institute, Kyoto University. Throughout the entire experiments, the animals were kept in individual cages that were placed inside a special safety cabinet. The room temperature (23–26 °C for macaque monkeys and rats; 26–30 °C for marmosets) and the light condition (12-hr on/off cycle) were controlled. The animals were fed usually with dietary pellets and had free access to water. Every effort was made to minimize animal suffering.

### Viral vector production

The construction of envelope plasmids (pCAGGS-FuG-B2 and pCAGGS-FuG-E) was mentioned elsewhere^17,19^. The transfer plasmid (pCL20c-MSCV-cgfTagRFP) contained the cDNA encoding cysteine- and glycan-free (cgf) TagRFP^39^ under the control of the murine stem-cell virus (MSCV) promoter. DNA transfection and viral vector preparation were carried out as previously described^19,29^. Briefly, HEK293T cells in 72 10-cm tissue culture dishes were transfected with transfer, envelope, and packaging plasmids (pCAG-kGP4.1R and pCAG4-RTR2) by using the calcium-phosphate precipitation method. Eighteen hours after transfection, the medium was exchanged to a fresh one, where the cells were incubated for 24 hr. The medium was then harvested and filtered through a 0.45-µm Millex-HV filter unit (Millipore, USA). Viral vector particles were collected by centrifugation at 6,000 × g for 16–18 hr and resuspended in 0.01 M phosphate-buffered saline (PBS). The particles were applied to a Sepharose Q FF ion-exchange column (GE Healthcare, UK) in 0.01 M PBS and eluted with a linear <1.5 M NaCl gradient. The fractions were monitored at 260/280 nm of absorbance wavelength. The peak fractions containing the particles were collected and condensed to 110 µl by centrifugation through a Vivaspin filter (Vivascience, UK).

To measure RNA titer, viral RNA in 50 nl of the vector stock solution was isolated by using a NucleoSpin RNA virus kit (Takara, Japan), and the copy number of the RNA genome was determined by quantitative PCR with Taq-Man technology (Thermo Fisher Scientific, USA). The viral protein signals of the HiRet vector (4.0 × 10^6^ genome copies) and the NeuRet vector (4.0 × 10^6^ and 1.6 × 10^7^ genome copies) were visualized by 4–12% SDS-acrylamide gel electrophoresis and fluorescent staining (Oriole, BIO-RAD, USA). HEK293T and Neuro2A cells were transduced with the viral vectors (100 genome copies/cell), and stained with Hoechst 33342 (Thermo Fisher Scientific, USA). The transducing unit was estimated by flow cytometry (FACSCanto II, BD Biosciences, USA). The RNA copy number of each vector was divided by the transduction unit to calculate the ratio of the RNA copy number to transducing units in each five individual experiment.

### Surgical procedures

The macaque monkeys were initially sedated with ketamine hydrochloride (5 mg/kg, i.m.) and xylazine hydrochloride (0.5 mg/kg, i.m.), and then anesthetized with sodium pentobarbital (20 mg/kg, i.v.). During the surgery, the monkeys were kept hydrated with a lactated Ringer’s solution (i.v.). An antibiotic (Ceftazidime; 25 mg/kg, i.v.) and an analgesic (Meloxicam; 0.2 mg/kg, s.c.) were applied at the initial anesthesia. After removal of a portion of the skull, multiple injections of each vector were made unilaterally into the striatum by means of a magnetic resonance imaging (MRI)-guided navigation system (Brainsight Primate, Rogue Research, Canada). In total, 60 µl of the FuG-B2 (2.5 × 10^10^ genome copies/ml) or FuG-E (2.5 × 10^10^ or 7.5 × 10^10^ genome copies/ml) pseudotyped lentiviral vector was injected through a 10-µl Hamilton microsyringe into both the caudate nucleus and the putamen at six rostrocaudal levels (3–5 µl/site, two sites per track, eight tracks per monkey; three tracks for the caudate nucleus and five tracks for the putamen). After the injections, the scalp incision was sutured. The monkeys were monitored until the full recovery from the anesthesia.

The marmosets were initially sedated with ketamine hydrochloride (50 mg/kg, i.m.) and xylazine hydrochloride (2 mg/kg, i.m.), and then anesthetized with sodium pentobarbital (15 mg/kg, i.p.). An antibiotic (Cefmetazole; 25 mg/kg, i.m.) and 10 ml of acetated Ringer’s solution (s.c.) were administrated before and after the operation. After the craniotomy, multiple injections of each vector were made unilaterally into the striatum of which coordinates were measured by using MRI. In total, 10 µl of the FuG-B2 or FuG-E pseudotyped lentiviral vector (each 1.0 × 10^10^ genome copies/ml) was injected through a 10-µl Hamilton microsyringe into both the caudate nucleus and the putamen at three rostrocaudal levels (1 µl/site, two sites per track, five tracks per marmoset; three tracks for the caudate nucleus and two tracks for the putamen). After the injections, the scalp incision was sutured, and an analgesic (Meloxicam; 1 mg/kg, s.c.) was applied.

The rats were anesthetized with a combination of ketamine hydrochloride (50 mg/kg, i.m.) and xylazine hydrochloride (4 mg/kg, i.m.). In total, 2 µl of the FuG-B2 or FuG-E pseudotyped lentiviral vector (each 1.0 × 10^10^ genome copies/ml) was stereotaxically injected into the right dorsal striatum (0.5 µl/site, four sites) through a glass microinjection capillary connected to a microinfusion pump. The anteroposterior, mediolateral, and dorsoventral coordinates (mm) from the bregma and dura were 1.5/2.8/5.8, 1.5/2.8/4.0, 0.0/3.5/5.5 and 0.0/3.5/4.0 for the dorsal striatum according to an atlas of the rat brain^40^.

### Immunohistochemistry

After a survival period of four weeks, the animals were deeply anesthetized with an overdose of sodium pentobarbital (50 mg/kg, i.v., for macaque monkeys; 50 mg/kg, i.p., for marmosets; 100 mg/kg, i.p., for rats) and perfused transcardially with 0.1 M PBS, followed by 10% formalin in 0.1 M phosphate buffer. The removed brains were postfixed in the same fresh fixative overnight at 4 °C, and saturated with 30% sucrose in 0.1 M PBS at 4 °C. A freezing microtome was used to cut coronal sections serially at the 50-µm thickness for macaque monkeys and the 40-µm thickness for marmosets and rats. Every tenth section was mounted onto gelatin-coated glass slides and Nissl-stained with 1% Cresyl violet.

Immunoperoxidase staining for tRFP was performed as described previously^29^. The sections were pretreated with 0.3% H_2_O_2_ for 30 min, rinsed three times in 0.1 M PBS, and immersed in 1% skim milk for 1 hr. Then, the sections were incubated for 2 days at 4 °C with rabbit polyclonal anti-tRFP antibody (1:2,500 dilution; Thermo Fisher Scientific) in 0.1 M PBS containing 2% normal donkey serum and 0.1% Triton X-100. The sections were subsequently incubated with biotinylated donkey anti-rabbit IgG antibody (1:1,000 dilution; Jackson laboratories, USA) in the same fresh medium for 2 hr at room temperature, followed by the avidin-biotin-peroxidase complex kit (ABC Elite; 1:200 dilution; Vector laboratories, USA) in 0.1 M PBS for 2 hr at room temperature. The sections were reacted for 10–20 min in 0.05 M Tris-HCl buffer (pH 7.6) containing 0.04% diaminobenzine tetrahydrochloride (Wako, Japan), 0.04% NiCl_2_, and 0.002% H_2_O_2_. The reaction time was set to make the density of background immunostaining almost identical. These sections were counterstained with 0.5% Neutral red, mounted onto gelatin-coated glass slides, dehydrated, and coverslipped.

For double immunofluorescence histochemistry for RFP and one of NeuN, GFAP, and TH, the sections were immersed in 1% skim milk for 1 hr and incubated for 2 days at 4 °C with rabbit polyclonal anti-tRFP antibody (1:1,000 dilution; Thermo Fisher Scientific) and mouse monoclonal antibodies; anti-NeuN antibody (1:2000 dilution; Millipore, USA), anti-GFAP antibody (1:500 dilution; Sigma, USA), and anti-TH antibody (1:500 dilution; Millipore). The sections were subsequently incubated for 2 hr at room temperature with a cocktail of Cy3-conjugated donkey anti-rabbit IgG antibody (1:400 dilution; Jackson laboratories) and Alexa 647-conjugated donkey anti-mouse IgG antibody (1:400 dilution; Jackson laboratories) in the same fresh medium. For double immunofluorescence histochemistry for Iba1 and CD8, rabbit monoclonal anti-Iba1 antibody (1:1,000 dilution; Wako), mouse monoclonal anti-CD8 antibody (1:1,000 dilution; Bio-Rad, USA), Alexa 488-conjugated donkey anti-rabbit IgG antibody (1:200 dilution; Jackson laboratories), and Alexa 647-conjugated donkey anti-mouse IgG antibody (1:200 dilution; Jackson laboratories) were used following the staining protocol described above.

### Image acquisition and histological analyses

To capture brightfield microscopic images, an optical microscope equipped with a high-grade charge-coupled device (CCD) camera (Biorevo, Keyence, Japan) and a scientific CMOS camera (In Cell Analyzer 2200, GE Healthcare) were used. A confocal laser-scanning microscope (LSM800, Carl Zeiss, USA) were used to take fluorescent microscopic images. The number or density of RFP-positive cells in each brain region were calculated with Neurolucida software (MicroBrightField, USA) and Matlab software (Mathworks, USA), as previously described^29^. For counts of RFP-positive cells in the SNc, 11, 7, or 6 sections were used in macaque monkeys (500-μm apart), marmosets (400-μm apart) or rats (320-μm apart), respectively. For counts of RFP-positive cells in the CM-Pf or Pf thalamic nucleus, 7, 4, or 4 sections were used in macaque monkeys, marmosets or rats, respectively. For measurements of the density of RFP-positive cells in the cortical areas, 10, 8, or 4 sections were used for the macaque SMA in macaque monkeys, area A6m in marmosets, or M1 in rats, respectively. Stereological cell counting assisted with StereoInvestigator software (MBF Biosciences, USA) was performed to estimate the ratio of RFP + NeuN-positive or RFP + GFAP-positive cells to the total RFP-positive cells at the injection sites and the ratio of RFP-positive cells to the total TH-positive cells in the SNc. Labeled cells in 100-μm × 100-μm counting frames equally spaced across a 500-μm × 500-μm grid were counted with an 18-μm-high optical dissector.

### Statistics

Values were expressed as the mean ± SEM of the data. For statistical comparisons, Student’s *t* tests were used with significance set at *P < 0.05, **P < 0.01, or ***P < 0.001.

## Supplementary information

Supplementary Information
