# Supplementary material for: A note on retrograde gene transfer efficiency and inflammatory response of lentiviral vectors pseudotyped with FuG-E vs. FuG-B2 glycoproteins
Source: Sci Rep. 2019 Mar 5;9:3567. doi: 10.1038/s41598-019-39535-1 (PMC6400974; doi:10.1038/s41598-019-39535-1)
Supplement: Supplementary file 1 — Supplementary Information [file 41598_2019_39535_MOESM1_ESM.pdf]

SUPPLEMENTARY INFORMATION

A note on retrograde gene transfer efficiency and inflammatory response of lentiviral vectors pseudotyped with FuG-E vs. FuG-B2 glycoproteins

Soshi Tanabe, Shiori Uezono, Hitomi Tsuge, Maki Fujiwara, Miki Miwa, Shigeki Kato, Katsuki Nakamura, Kazuto Kobayashi, Ken-ichi Inoue, Masahiko Takada

Supplementary Figure S1.

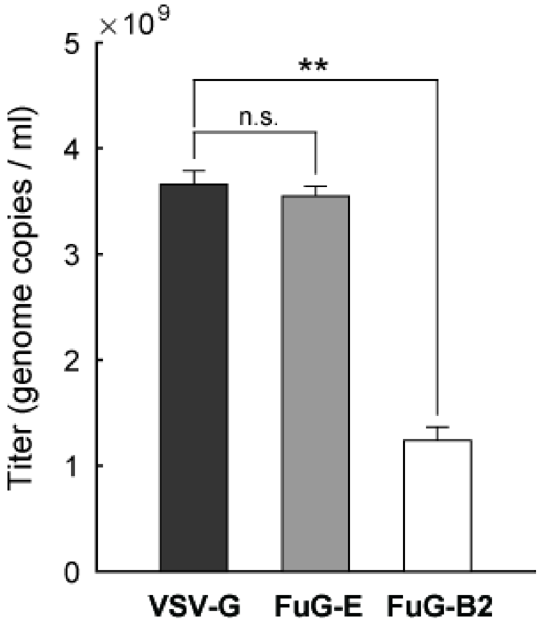

Supplementary Figure S1. Production efficiency of lentiviral vectors pseudotyped with VSV-G, FuG-E, and FuG-B2 in smaller-scale production.

HEK293T cells in two 10-cm tissue culture dishes were transfected with the envelope plasmid encoding VSV-G, FuG-E, or FuG-B2, together with the transfer plasmid encoding GFP and packaging plasmids. Vector particles were concentrated by centrifugation and suspended in 200 μl of PBS. The yield in the vector stock solutions measured by quantitative reverse transcription-PCR analysis (filled bar for VSV-G, gray bar for FuG-E, and open bar for FuG-B2). Each value was obtained from four individual experiments and expressed as the mean ± SEM. $**P < 0.01$, significant difference from the VSV-G vector (Bonferroni test). n.s., not significant.

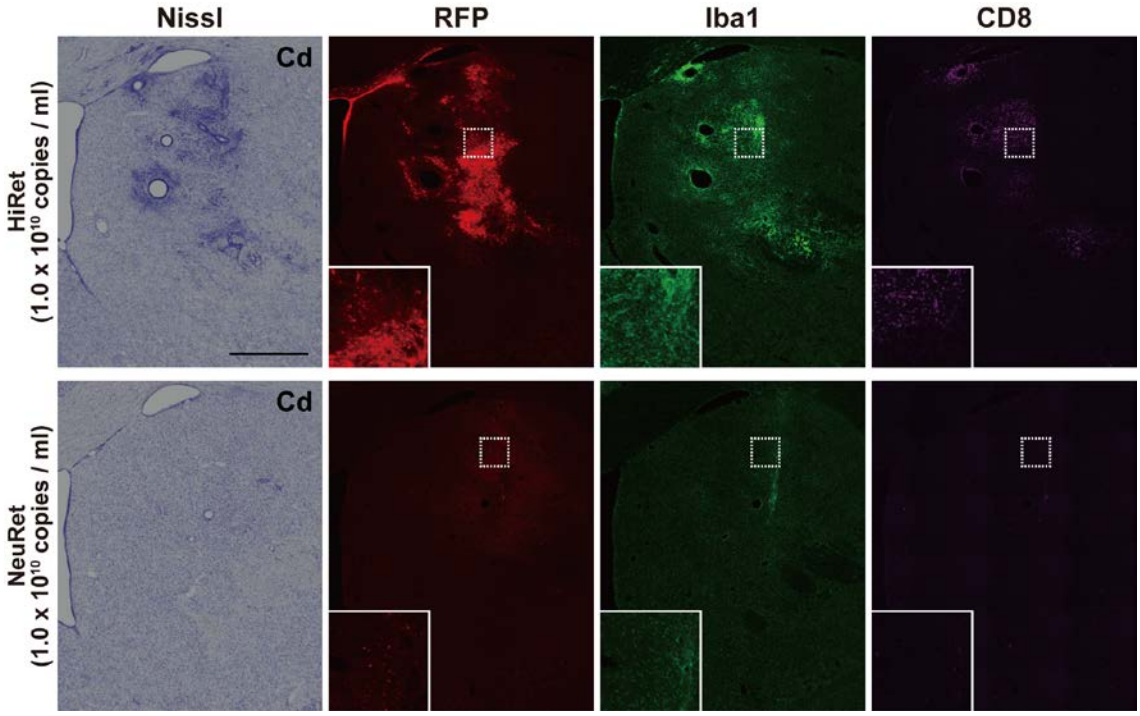

Supplementary Figure S2. Inflammatory responses after intrastriatal injections of the HiRet and NeuRet vectors in marmosets.

Nissl staining, RFP-native fluorescence (red), and immunofluorescent staining for Iba1 (green) and CD8 (magenta) at the injection sites of the HiRet (upper) and NeuRet (lower) vectors in marmosets. Cd, caudate nucleus. Scale bar, 500 μm.

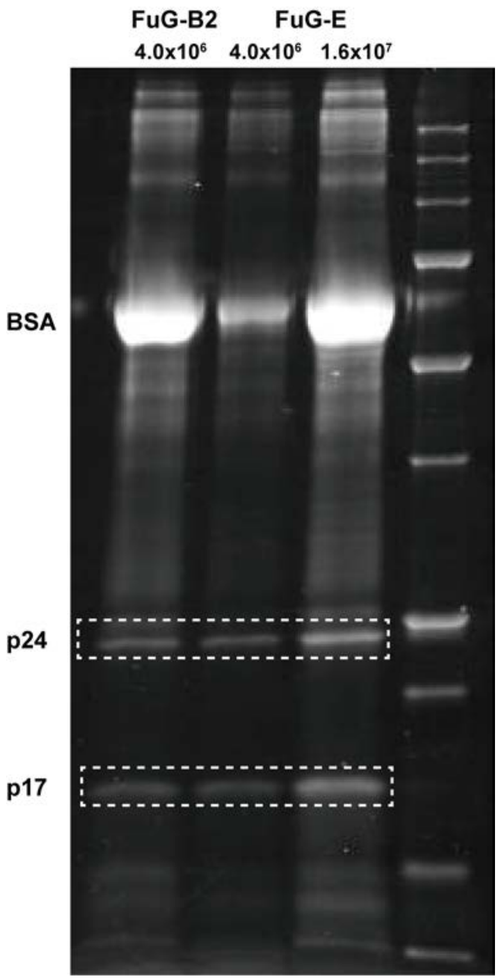

Supplementary Figure S3. Full-length gel image of Figure 1b.

Original SDS-PAGE image of Figure 1b. White dotted lines indicate bands of the viral protein (p24 and p17). BSA, bovine serum albumin.
